# The Evolution of Our Understanding of Immunoproliferative Small Intestinal Disease (IPSID) over Time

**DOI:** 10.3390/curroncol29050301

**Published:** 2022-05-23

**Authors:** Ruah AlYamany, Mohamed A. Kharfan-Dabaja, Mehdi Hamadani, Alfadel Alshaibani, Mahmoud Aljurf

**Affiliations:** 1 Section of Hematology and Bone Marrow Transplant, King Faisal Specialist Hospital and Research Center, Riyadh 11564, Saudi Arabia; aalshaibani@kfshrc.edu.sa (A.A.); maljurf@kfshrc.edu.sa (M.A.); 2 Division of Hematology-Oncology, Blood and Marrow Transplantation Program, Mayo Clinic, Jacksonville, FL 32224, USA; KharfanDabaja.Mohamed@mayo.edu; 3 BMT and Cellular Therapy Program, Department of Medicine, Medical College of Wisconsin, Milwaukee, WI 53226, USA; mhamadani@mcw.edu

**Keywords:** immunoproliferative small intestinal disease (IPSID), lymphoma, α-heavy chain disease

## Abstract

Immunoproliferative small intestinal disease (IPSID) is an uncommon disease with a higher prevalence in the developing world. IPSID diagnosis relies mainly on a tissue biopsy and a high index of suspicion. Treatment options are variable; however, they mainly include anthracycline-based chemotherapy with or without antibiotics in advanced stages. Because of the paucity of IPSID, our perception of the disease remains narrow, and investigating the optimal lines of therapy and prevention without a complete comprehension of the disease is challenging. In our review, we explore the expansion of knowledge about IPSID, which has been developing over the years, to help increase the detection of IPISD cases and further research the most appropriate lines of therapy and prevention.

## 1. Introduction

Immunoproliferative small intestinal disease (IPSID) represents a spectrum of indolent lymphoproliferative syndrome that involves different clonal disorder stages. It is considered a type of extra-nodal marginal zone B-cell lymphoma (MZL) of mucosa-associated lymphoid tissue (MALT) [1,2]. 

Marginal zone lymphomas originate from the follicular marginal zone in the lymph nodes and are classified based on their anatomical involvement in three subtypes: splenic MZL, nodal MZL, and extra-nodal MZL [1].

IPSID was first recognized in the early 1960s when it was termed small intestinal lymphoma; it was first described in the Mediterranean region and was previously known as “Mediterranean lymphoma.” [1]. Later in that decade, patients with Mediterranean lymphoma were noted to have abnormal IgA molecules in their body fluids and serum. These molecules were further studied and found to have a truncated alpha heavy chain; hence, the name was changed to alpha heavy chain disease [1,3].

In 1978, the World Health Organization (WHO) further evaluated the disease and recognized that it was not necessarily a pre-lymphoma stage, but more of a spectrum of diseases, which included alpha heavy chain disease and Mediterranean lymphoma with different stages: benign, intermediate, and malignant. Furthermore, a more descriptive term was given, namely IPSID [1,4].

## 2. Epidemiology

IPSID is not common in the Western world. The majority of cases, mostly those described early on, were found in the Mediterranean region with many patients being of Arab and non-European Jewish ancestry. Later, IPSID was identified in other areas, including south and central Africa and Southeast Asia. More recently, sporadic cases have been acknowledged in parts of Europe and the American continent [1,5,6,7]. It is unclear, however, if those cases are the result of migration.

Risk factors that have been commonly associated with the development of IPSID included low socioeconomic status, poor sanitation, and clean water inaccessibility, which might have led to a higher incidence of chronic or recurrent gastrointestinal infections and possibly, at least initially, partially explained the association between IPSID and developing countries [1,5,6,8].

With improved sanitation techniques and the availability of clean water, a decreased incidence of IPSID has been described in those areas considered at a higher risk [7,9].

An underlying genetic predisposition is suggested, with evidence of human leukocyte antigen (HLA) association, a pattern of elevated alkaline phosphatase noticed in the healthy family members of the affected individuals, and defective cellular and humoral immunity. However, these findings are not considered sufficient evidence of a clear genetic association, highlighting that environmental factors alone are not solely responsible for the occurrence of IPSID [3,5,6]. There have been reports on the association of IPSID with HLA-AW19, HLA-A9, HLA-B12, and the B blood group [3]. 

The majority of patients with IPSID appear to be diagnosed in their second and third decades of life. An estimated ratio of male-to-female incidence of 2.4:1 has been described in some reports [1,2].

## 3. Etiology and Pathophysiology

**Figure 1:**
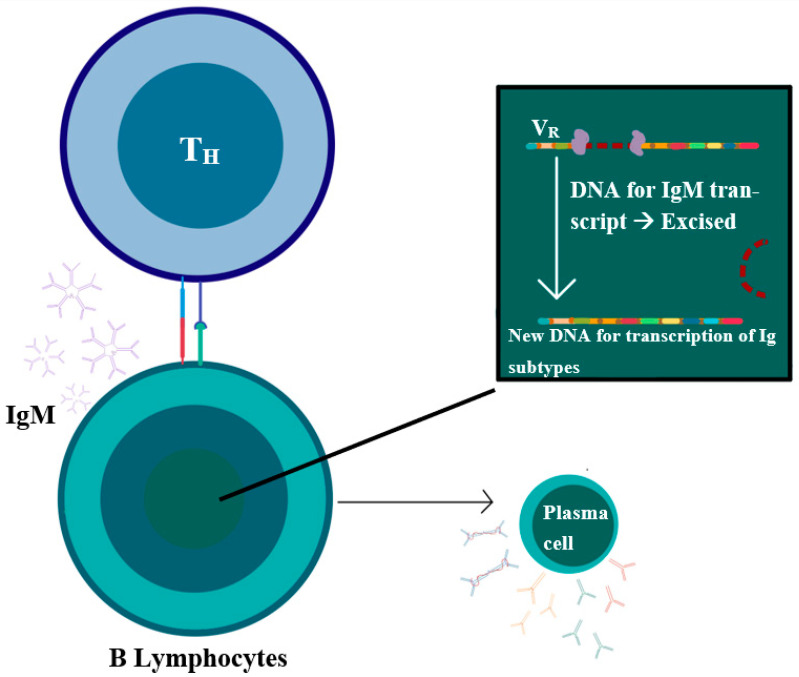
T helper cells bind to B lymphocytes through CD40L and TCR to the MHC-II and CD40 receptors on the B lymphocytes, promoting different steps responsible for the variable functions of B-cells, including (1) excision of the DNA responsible for IgM transcription and the attachment of the variable regions (V_R_) to the rest of the DNA leading to the production of different classes of immunoglobulin, a process also known as class-switching. (2) Somatic hypermutation, which is responsible for increasing the antigen-binding specificity. (3) Formation of the germinal center.

Generally, upon exposure to antigens, B lymphocytes bind to TH1 cells and undergo somatic hypermutations to increase antigen-binding specificity and immunoglobulin class-switching from IgM production to the production of IgG, IgA, and IgE subtypes of immunoglobulins (Figure 1). IPSID is potentially associated with a chronic inflammatory state, where there is obstinate antigen stimulation, either from persistent infections or other possible inflammatory conditions, such as an autoimmune disease. This persistent stimulation leads to the proliferation of lymphoid cells and the production of increased amounts of IgA immunoglobulins, which typically undergo somatic hypermutations to produce antigen-specific immunoglobulins. However, in IPSID, the persistent antigen presentation with ongoing somatic hypermutations can lead to a clonal production of an abnormal IgA with an abnormal α-heavy chain (α-HC) protein, which lacks the variable heavy chain (V_H_), and the first constant domain (C_H_1), which is present on the light chain segment of the immunoglobulin [3].

**Figure 2:**
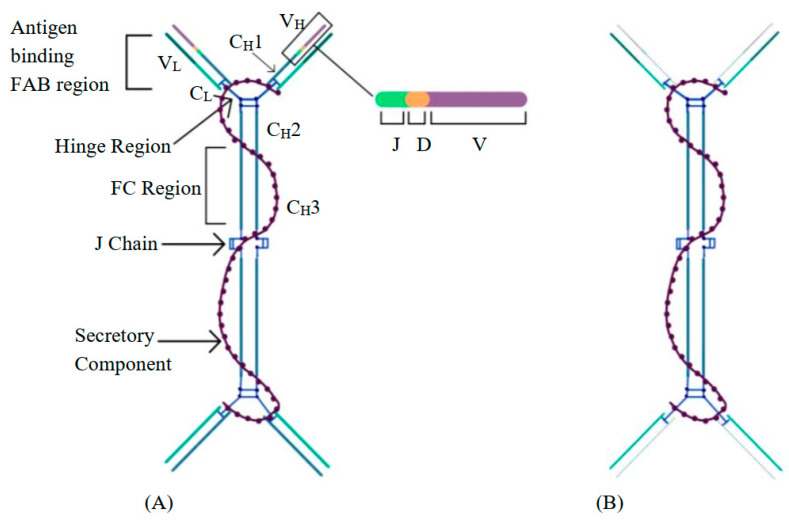
(**A**) Normal IgA structure. (**B**) Aberrant IgA molecule with missing V_H_ and C_H_1 regions.

The defective α-HC protein loses the ability to bind the light chain, which leads to an abnormal configuration of the produced immunoglobulin [10] (Figure 2).

This aberration results from ongoing mutations and alternative splicing along with the abnormally short α-HC mRNA, which results in an abnormal α-HC protein containing various in-frame inserts between the second and third constant regions and the leader peptides, hence producing an unrecognized non-human genomic material [1,3,11].

The hallmark of IPSID is cell clonality, which has been reported at all stages [6,10]. This clonality suggests that IPSID is lymphomatous at all stages, but it does not always progress to aggressive lymphoma because the cell’s normal growth regulators can perhaps balance out the clonality. However, the balance is apparently lost once further genetic modifications occur and anti-apoptotic effects are enhanced. The cells widely proliferate irrepressibly with minimal or no apoptosis, resulting in the accumulation of lymphoid cells and progression to full-blown lymphoma [6,12].

IPSID shares features with other lymphoproliferative diseases, including MALT lymphoma, lymphoplasmacytic lymphoma, and plasma cell neoplasms [3]. Both IPSID and MALT lymphoma develop from antigen-driven B lymphocyte activation, which results in clonal disease and later malignant transformation [4]. Based on the similarities between MALT and IPSID and the association of MALT lymphoma with H. Pylori, some suggested infectious agents may be responsible for the chronic antigen stimulation in IPSID, including small intestinal bacterial overgrowth, giardiasis, and parasitic infections; trichuriasis [1,2,3,8]. Specific bacterial organisms linked to IPSID on different occasions include Campylobacter jejuni (most common), Vibrio cholera, Vibrio fluvialis, Escherichia coli, and H. pylori, although there is minimal evidence of association with the latter [1,2,3,4,6].

Despite the multiple reported findings of C. jejuni-positive cultures in patients with IPSID, the exact mechanism is unknown and definite proof of a seldom association between C. jejuni and IPSID remains unknown. Moreover, it is not clear whether there is a direct association between IPSID and the aforementioned organisms or if it is a mere coincidence due to the poor sanitation setting that IPSID has been associated with [1]. C. jejuni is hypothesized to be able to generate a mucosal response with the secretion of IgA at high levels, which creates a persistent stimulation and clonal α-heavy chain secretion with no antigen–antibody Fc-dependent suppression [13].

AlSaleem et al. hypothesized that patients with IPSID, with evidence of C. jejuni infections, might have had a previous infection with V. cholerae, especially since the 1960s cholera epidemic occurred in the same geographic district that IPSID was commonly diagnosed in a couple of years after. The V. cholerae toxin is known to break down the double-stranded DNA, which alters the structure and causes mutations involving the PAX5 gene (encodes typically for the transcription factor B-cell specific activator protein (BSAP)) and other oncogenes in the B lymphocytes, which lead to abnormal lymphopoiesis and the production of the aberrant IgA, and later progress to lymphoma [3].

The intestinal wall, which creates a barrier between the human body and the external environment, comprises three layers: the outer, middle, and internal layers. The outer layer is composed of the mucous layer, intestinal microbiota, and defense proteins (antimicrobial proteins and IgA). The middle layer is mainly composed of intestinal epithelial cells and, lastly, the inner layer is composed of the innate and adaptive immune cells [14]. It is believed that many intestinal inflammatory conditions are caused by interruption of the intestinal barrier and microbial dysbiosis. This intestinal alteration and overgrowth of specific organisms are thought to contribute to the pathophysiology of IPSID, especially in the early stages, where a complete response is seen with the use of antibiotics alone [15].

## 4. Clinical Features and Prognosis

At presentation, common symptoms include non-bloody watery, copious diarrhea, low-grade fevers, night sweats, abdominal pain, weight loss, steatorrhea, nausea, and vomiting, which can lead to malnutrition and electrolytes imbalances manifesting as carpopedal spasms and tetany, among others [1,3,5,8,10,16]. In some patients, fasting appears to decrease symptoms, while in others, consumption of lactose-containing products is associated with worsening symptoms [4,11].

Rarely, melena and other forms of gastrointestinal bleeding can occur, with positive occult blood in stool testing. Amenorrhea can be present, possibly as a consequence of severe malnutrition [1,13].

A physical examination reveals signs of malnutrition and defective absorption, including cachexia, peripheral edema, and finger clubbing, among others. Abdominal distention and tenderness can be present with or without a palpable abdominal mass [1,10]. Spreading of the disease beyond the abdomen, including palpable peripheral lymphadenopathy, hepatosplenomegaly, and bone marrow involvement, are not commonly seen in IPSID; however, they can be present (rarely) in advanced stages of the disease [1,3,5].

If IPSID is left untreated, it can progress and transform into higher grades of B-cell lymphomas and lead to further malabsorption and malnutrition. Other complications seen with the advanced disease include infections, intussusception, intestinal obstruction, gastrointestinal bleeding, and perforation, resulting in severe morbidity and mortality [1,3,17]. Extra-intestinal complications are not common, but reports have described nephropathies, osteoarthropathy, and osteomalacia [1]. The rate of progression of IPSID to high-grade lymphomas is not well described [3].

Most patients are diagnosed when these manifestations develop, and in retrospect, patients frequently report mild symptoms dating back five to ten years preceding the clinical presentation, with an average duration of symptoms lasting between one month to 6 years [3,5,11]. The natural course of the disease is highlighted with episodic manifestations with intervals of improvement in between, likely contributing to a delay in the diagnosis [1].

## 5. Differential Diagnoses

The most important factor in diagnosing IPSID is having a high index of suspicion, especially in patients with unexplained chronic diarrhea from endemic parts of the world [4,18]. The presentation of IPSID is very similar to other infectious and inflammatory gastrointestinal conditions, e.g., celiac disease and chronic intestinal infections, tropical sprue, AIDS enteropathy, intestinal tuberculosis, inflammatory bowel disease, and Whipple’s disease [1,2,10,16,18]. Hence, it can be challenging to differentiate between these diseases and IPSID; yet some features might favor one diagnosis over the other. For instance, IPSID is usually more intense with involvement of the whole small intestine at times less responsive to a gluten-free diet compared with celiac disease, and stool cultures can be negative, unlike intestinal infections.

Histologically, IPSID and celiac disease both have surface epithelial damage, villous atrophy, and mucosal infiltrates with lympho-plasmacytes; however, IPSID has atrophic crypts, and hyperplastic elongated crypts are seen in celiac disease [2,10,16,18]. Furthermore, serum antibodies to tissue transglutaminase (anti-TTG) positivity help rule in celiac disease while abnormal serum protein electrophoresis would favor IPSID. The development of high-grade lymphomas can complicate both IPSID and celiac disease, yet in the case of celiac disease, if progression to lymphomas ensues, those are T-cell lymphomas and are usually prone to perforation. In the case of IPSID, the disease generally progresses to B-cell lymphomas and it is less likely to present with perforations [10].

Tropical sprue does not commonly present with abdominal pain and usually has an abnormal D-xylose test [18]. We acknowledge that differentiating between IPSID and chronic inflammatory gastrointestinal conditions with clinical manifestations and pathology alone might be challenging. Studies to evaluate the heavy chain immunoglobulin (IgH) gene rearrangement and immunohistochemical stains to confirm the expression of heavy chains only from plasma cells can be helpful in such situations [2,10].

## 6. Diagnostics and Investigations

Reported cases of IPSID were diagnosed between 1 month and 6 years from the initial presentation, with a median time for diagnosis of approximately 10 months [1,16]. Most reported cases had similar laboratory (Table 1), histopathological, and radiological features.

### 6.1. Radiological Findings

Most studies illustrated the findings seen in barium X-ray and CT studies. One report conveyed the findings of an abdominal ultrasound, which showed peripancreatic lymphadenopathy with oval-shaped hypoechoic regions [4].

Barium X-ray studies were done as part of routine work in the old literature, and the reported transit time was 3–8.5 h with a median of 4.5 h [19]. Findings included small intestinal diffuse dilatation, strictures, infiltrative, nodular defects, intussusception, and mucosal fold thickening of various degrees with irregular edges; “Postage stamp” sign, and sprue-like changes [4,19,20]. Secretory assessments varied between hypersecretion in most cases, but some had dry malabsorption, similar to findings seen in amyloidosis. An ominous sign that suggests progressive stages of lymphoma is the appearance of widening and displacement of the bowel loops [19].

Computed tomography (CT) of the abdomen commonly shows mesenteric and retroperitoneal lymphadenopathy with different degrees of involvement depending on the radiological stage, as described by Vessal et al. (Table 2) [19]. Diffuse small bowel mural thickening with the presence of pseudo-polyps with strictures and segmentations, most commonly in the proximal part of the small intestine, are most prevalently in the jejunum [1,3,5,8,10,21]. The presence of organomegaly has not been commonly documented. Features suggestive of osteomalacia have been noted due to malabsorption [5].

PET-CT featured hypermetabolic FDG activity involving the small bowel wall and mesenteric lymph nodes [1,8].

**Table 1:** Investigational features reported on in the literature in patients diagnosed with IPSID.

Chemistry	Electrolyte imbalances (hypokalemia, hyponatremia, hypocalcemia, non-anion gap metabolic acidosis) [1,8,21].
	Low albumin.
	Elevated alkaline phosphatase.
	Hypocholesteremia [5].
	Vitamin deficiencies (vitamin B12 and folate) [5].
	Elevated lactate dehydrogenase (LDH), reported in advanced stages of lymphoma [11].
Hematology [1,8]	Complete blood count: mild anemia and leukocytosis.
	Peripheral blood morphology can demonstrate plasmacytic infiltrates.
	Bone marrow biopsy rarely is involved and can show plasmacytosis and features of plasma cell leukemia [20].
Microbiology [1,8,12,21]	Stool cultures were stated in different reports to be positive for various organisms including Campylobacter jejuni, Vibrio fluvialis, Giardia.
	Tissue culture was positive for E. coli in one case report.
Immunology and Electrophoresis	Low or normal IgA levels with normal-to-high IgG and IgM levels [4].
	No Bence-Jones proteins in the urine [4].
	Decreased or absent cellular and humoral responses.
	Immuno-electrophoresis and immunoselection detect α-heavy chain proteins in serum and body fluids, it is considered the most sensitive and specific method and is detected in almost 70% of cases [4,11].
Other	Positive stool occult [1].
	Sudan III stain of the stool positive, suggestive of malabsorption [18].

**Table 2:** Vessal et al. radiological staging of IPSID.

Radiological Stage	Description
Stage I	Focal lymphoma involving the mucosa or submucosa or mesenteric lymph node.
Stage II	Lymphoma extension to the transmural layer and lymphadenopathy in several regions.
Stage III	Lymphoma involvement of the bowel and massive mesenteric lymph nodes.
Stage IV	Lymphoma involving the bowel and extra-mesenteric lymph nodes or parenchymatous organs.

### 6.2. Esophagogastroduodenoscopy and Laparotomy 

Endoscopies and laparotomy with full-thickness biopsies at a minimum of three different sites with lymph node biopsies are optimal diagnostic tools, along with immunoselection and electrophoresis to confirm IPSID [3,4,12,18]. Patterns often reported include disseminated erythema with atrophic villi and nodular mucosa, ulcerations, small intestinal wall thickening, lymphangiectasis, cobble-stoning appearance, and mesenteric lymphadenopathy [1,8,21].

Ulcerations can be seen at any stage, but usually indicate a malignant process [4]. Intestinal masses and dilation were seen on laparotomy in some patients, while others had normal-appearing bowels [10]. The majority of findings were found in the small intestine, mainly in the proximal part; some reviews reported the jejunum to be the most commonly affected site. Gastric and clonal mucosa are usually spared [8].

### 6.3. Histopathology and Immunohistochemistry Stains

Under the microscope, small intestinal villous blunting and shortening with absent crypts and mucosal flattening is seen in patients with IPSID [3,21]. These changes are due to infiltration of the lamina propria with lymphoplasmacytic cells, which secrete the abnormal monoclonal α-HC protein and cause disjunction of the Lieberkühn crypts [1,2,4]. The surface epithelium is usually intact, particularly in the early stages [1]. The earliest pathological feature is the presence of abundant infiltrates of centrocyte-like lymphocytes and plasma cells on the intestinal biopsy; it was previously referred to as stage 0 of IPSID [22].

Mucosal infiltrates can be of four different types: diffuse lymphoplasmacytic (DLP), which is commonly benign. Follicular lymphoid (FL) infiltration can be follicular hyperplasia or lymphoma, mixed cellularity of both FL in the superficial mucosa and DLP patterns in the deeper layers; most of these patients were documented as having follicular lymphoma. Lastly, the non-specific type usually causes continuous longitudinal infiltrates with increased cellularity of normal-appearing lymphocytes, plasma cells, and eosinophils [7].

In later stages, atypical lymphoid cells with speckled immunoblastic cell aggregates develop and can progress into sheets of dystrophic plasma cells that invade the submucosa and muscularis propria. These cells seem to be interconnected to the development of neoplasm [3,4,12]. Moreover, a predominance of mature plasma cells on the sample could be indicative of a hidden lymphoma in the deeper layers or as a predictive feature of the future development of lymphoma [20]. Lesions with different stages of the disease can be present within the same part of the intestine [20].

Based on histological features alone, two variants of IPSID have been described [18].

1. Diffuse infiltration by plasma cells solely or mixed with lymphocytes in the intestine’s mucosa at sites other than the neoplastic mass if present. The development of immunoblastic malignant lymphoma with detectable α-HC proteins in body fluids and tissues is commonly seen in association with this variant.
2. Diffuse follicular lymphoid hyperplasia in the mucosa of the small intestine. This variant is commonly associated with diffuse undifferentiated malignant lymphomas and undetectable α-HC proteins.

Galian et al. developed a comprehensive staging system for IPSID relying on the type of cellular infiltrates and mesenteric nodal involvement, classified into stages A, B, and C, in which appropriate treatment is based (Table 3) [2,23].

Immunohistochemistry staining shows monoclonal cytoplasmic defected α-heavy chains that lack light chains and the expression of CD20 positivity on tumor cells, CD138, CD79a, and IgA positivity on plasma cells, and no expressions of CD5 and CD10 on tumor cells [2,10].

### 6.4. Molecular and Cytogenetics

Although not frequently documented, some of the genetic abnormalities linked to IPSID include t(9;14), t(2;14), t(5;9), t(21;22) (q22;q11), 14q+ chromosome, abnormal p32 HC locus on chromosome 14 and light chain loci on chromosome 2 and 22. Mutations involving PAX5 genes were also reported [3,10]. Although IPSID shares multiple features with MALT lymphoma, the common translocation t(11;18) that is seen frequently in MALT-lymphoma is lacking in IPSID [3].

Clonal abnormalities of heavy chains are seen in most cases on molecular testing, even in the earliest phase [10].

## 7. Atypical IPSID Entities

Despite IPSID being an uncommon disease, there are still unique entities less commonly identified in the literature. These infrequent entities include non-secretory IPSID, an under-recognized subtype of IPSID distinguished from the typical secretory IPSID by the histological appearance rather than clinical manifestations, characterized by small centrocyte-like lymphoid cells without extensive plasmacytic differentiation that is usually seen in the secretory form [3].

Other rare forms of IPSID include gamma heavy-chain disease and α-HC disease of the colon, stomach, and lungs [3].

## 8. Management and Outcomes

The management of IPSID depends on the severity, histopathological features, and associated clinical manifestations. Treatment options include supportive measures, pharmacological agents, radiation therapy, and surgery. In the early stages, spontaneous remission has been described [3]; however, the disease can experience relapse with an advanced stage related to chronic antigenic stimulation; accordingly, early treatment is strongly recommended [3,24,25].

### 8.1. Supportive Measurements

Treatment complications of IPSID, including malnutrition, are required to facilitate the response to therapy and recovery of patients. Supportive measures need to consist of correcting unbalanced electrolytes, enhancing nutritional repletion with a high protein diet, and albumin replacement [20,21].

### 8.2. Pharmacological Therapy

**Figure 3:**
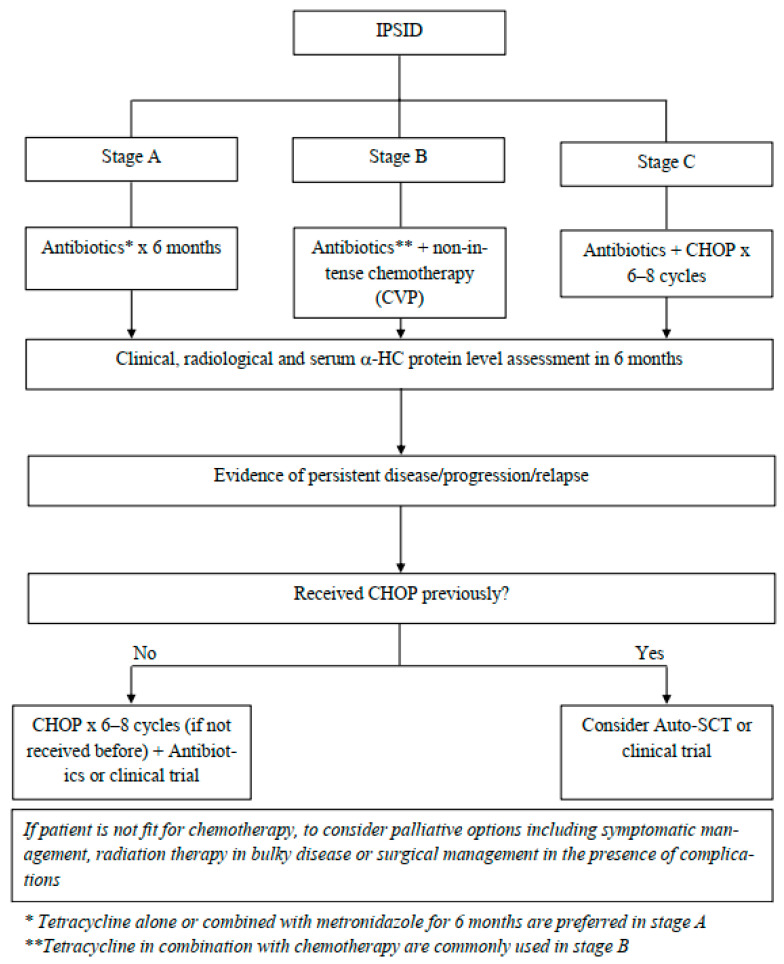
Suggested approach for management of IPSID stages and progression/relapse.

Therapeutic options depend on the stage of the disease, whether using antibiotics alone or in combination with chemotherapy; the therapeutic choice is commonly based on the Galian et al. staging system (Figure 3).

In the presence of early-stage disease, stage A, the choice of antibiotics as the only treatment modality is reasonable. Tetracycline alone or combined with metronidazole for 6 months was a popular regimen that resulted in clinical improvement, a reported 71% rate of complete remission, and a 5-year disease-free survival of 43% [3]. Other antimicrobial options include metronidazole with ampicillin, ciprofloxacin, doxycycline, and a 14-day course of piperacillin–tazobactam have been reportedly used to treat IPSID with observed clinical improvement [4,8,16].

Adjunctive steroids with antibiotics therapy can be of additional benefit in achieving a higher response rate in the early stages [4]. If no response is seen after completion of an antimicrobial therapy course after 6 months or if patients do not attain a CR in 1 year, repeating a biopsy is recommended, and consideration of starting chemotherapy appears reasonable [3,11].

As the disease progresses, the response to antibiotics diminishes, yet it can be used in intermediate stages, stage B, with or without chemotherapy. The suggested approach to stage B is using antibiotics, such as tetracycline with non-intense chemotherapy, for example, CVP (cyclophosphamide, vincristine, and prednisone), which has been reported to prolong remission [2,11].

Other reported treatment choices in intermediate stages include anthracycline-based chemotherapy or melphalan [3,11]. Further options include single chemotherapy (cyclophosphamide) with or without antibiotics and steroids, resulting in clinical, histological, and immunological remission [20].

In advanced stages, stage C, it is recommended to treat it as aggressive B-cell lymphoma with anthracycline-based chemotherapy and antimicrobial therapy with curative intent, traditionally with six–eight cycles of the CHOP regimen (cyclophosphamide, vincristine, doxorubicin, and prednisone), with a complete remission rate of 50–90% and a 3-year median survival rate of 67% [2,3,16,17]. Other available chemotherapy options include CHOP-bleomycin, m-BACOD (methotrexate, bleomycin, doxorubicin, cyclophosphamide, vincristine, and dexamethasone) [4].

The use of biological agents, such as rituximab, a monoclonal antibody against CD20, is not ascertained due to the presence of CD20-negative plasma cells but may be considered for CD20-positive lymphocytes. Further studies are needed to better understand the role of rituximab along with anti-myeloma therapeutic regimens [3,8]. There is no established role for prolonged maintenance with antibiotics [3].

### 8.3. Radiation Therapy

Involved site (abdominal) radiation can be used if a fast response is needed, particularly in bulky tumors to relieve symptoms [3].

### 8.4. Surgical Intervention

Surgery is usually recommended for selective management of complications, such as obstruction or perforation, and diagnosis and staging with the use of a laparotomy [3,20].

### 8.5. Stem Cell Transplantation

The role of an autologous stem cell transplant was recognized in patients with the refractory or relapsed disease [3]. The efficacy of an allogeneic stem cell transplant is not known, likely due to the low prevalence of the disease.

### 8.6. Response to Therapy and Maintenance Therapy

Response assessment is based on the improvement of clinical features with the elimination of diarrhea, and weight gain, radiologically by a PET-CT or CT abdomen with the resolution of uptake on PET, and diffuse thickening with regression of lymphadenopathy, along with histological normalization of small intestinal mucosal with the disappearance of monoclonal lymphoplasmacytic infiltrates. Measurement of the α-HC protein in the serum can be used as a marker for a response; however, this method has its limitations in terms of accuracy since (occasionally) the levels of α-HC proteins can regress in the presence of a transformation or relapse [3].

## 9. Conclusions

IPSID is an uncommon disease that is present in certain regions of the world, with the Middle East being one of the earliest (and most prevalent) areas from where cases have been reported. The key tool for diagnosing IPSID is having a high index of suspicion. The optimal treatment choice remains undefined, but the most agreed upon approach depends on the stage of the disease. Therapies include antibiotics alone or in combination with anthracycline-based chemotherapy regimens. Further descriptive studies and clinical trials are needed to establish the most effective therapeutic options. Further research is needed to thoroughly investigate the role of stem cells and targeted therapies in IPSID.

## Figures and Tables

**Table 3:** Galian et al. histopathological staging of IPSID.

Stage	Type of Cellular Infiltrate and Histopathological Description	Mesenteric Nodal Involvement
Stage A (Benign)	Heavy infiltrations of lamina propria with typical lymphocytes with few dysplastic plasma cells infiltrate with variable atrophic villi in the small intestine.	Few CD20-positive marginal zone B cells with plasmacytic infiltration of mesenteric or other abdominal and retroperitoneal lymph nodes with limited disorganization of histological structure.
Stage B (Intermediate)	Infiltration extending beyond the mucosa with atypical lymphoplasmacytic cells, immunoblastic-like cells with the areas remote from the mucosa, containing many dysplastic cells with total or subtotal villous atrophy.	Atypical plasmacytic and immunoblastic dense infiltrations causing structural changes of the mesenteric and abdominal lymph node construction.
Stage C (Malignant)	Immunoblastic high-grade B-cell lymphomas, some with strong CD20-positivity with plasmacytoid differentiation and proliferative histocytes extending into all layers of the intestinal wall and some forming confined large tumors of malignant formations.	Mesenteric and abdominal lymph nodes with sarcomatous proliferation alter the entire structure.

## Data Availability

Not applicable.
